# The heritable natural competency trait of *Burkholderia pseudomallei* in other *Burkholderia* species through *comE* and *crp*

**DOI:** 10.1038/s41598-018-30853-4

**Published:** 2018-08-20

**Authors:** Yun Heacock-Kang, Ian A. McMillan, Jan Zarzycki-Siek, Zhenxin Sun, Andrew P. Bluhm, Darlene Cabanas, Tung T. Hoang

**Affiliations:** 1 Department of Microbiology, University of Hawaii at Manoa, Honolulu, Hawaii USA; 2 Department of Molecular Biosciences and Bioengineering, University of Hawaii at Manoa, Honolulu, Hawaii USA

## Abstract

Natural competency requires uptake of exogenous DNA from the environment and the integration of that DNA into recipient bacteria can be used for DNA-repair or genetic diversification. The *Burkholderia* genus is unique in that only some of the species and strains are naturally competent. We identified and characterized two genes, *comE* and *crp*, from naturally competent *B*. *pseudomallei* 1026b that play a role in DNA uptake and catabolism. Single-copies of rhamnose-inducible *comE* and *crp* genes were integrated into a *Tn7* attachment-site in non-naturally competent *Burkholderia* including pathogens *B*. *pseudomallei* K96243, *B*. *cenocepacia* K56-2, and *B*. *mallei* ATCC23344. Strains expressing *comE* or *crp* were assayed for their ability to uptake and catabolize DNA. ComE and Crp allowed non-naturally competent *Burkholderia* species to catabolize DNA, uptake exogenous *gfp* DNA and express GFP. Furthermore, we used synthetic *comE* and *crp* to expand the utility of the λ-red recombineering system for genetic manipulation of non-competent *Burkholderia* species. A newly constructed vector, pKaKa4, was used to mutate the aspartate semialdehyde dehydrogenase (*asd*) gene in four *B*. *mallei* strains, leading to the complete attenuation of these tier-1 select-agents. These strains have been excluded from select-agent regulations and will be of great interest to the field.

## Introduction

Bacterial natural transformation, first described in 1928^1^, is the process in which exogenous DNA is taken from the environment by a recipient for nutrients, DNA repair, or genetic diversification^2^. Since then, this process has been described in 82 bacterial species including both gram-positive and gram-negative bacteria^3^. The molecular machinery that facilitates natural transformation is homologous to the type II secretion system (T2SS) and the type IV pilus (T4P)^2,3^. In gram-negative bacteria, DNA is transported across the outer membrane through the PilQ channel that houses the pseudopilus, PilE^3^, and is then shuttled across the periplasm by ComE^4^. An unknown nuclease generates a single-stranded DNA molecule that is transported into the cytoplasm through the ComA channel in the inner membrane^3^. The single-stranded DNA molecule is further broken down into nucleotide components or recombined into the chromosome of the recipient organism^2,5^. The ability of bacteria to be naturally transformable impart an evolutionary advantage and has driven diversification of species over time^2,6^.

*Burkholderia pseudomallei* (*Bp*) is the causative agent of the tropical disease melioidosis that presents in patients with diverse symptoms and clinical outcomes^7^. *Bp* is endemic to tropical regions around the world and readily isolated from the environment^8^. Different clinical and environmental isolates show a significant level of genetic diversity in part due to frequent recombination^9–11^. Clinical isolates have evolved within a host by removal of virulence loci and stress response regulators leading to asymptomatic infection^12^. Another example of recombination that occurred within the host is the evolution of a single *Bp* isolate into *B*. *mallei* (*Bm*), the causative agent of glanders^13^. Although glanders is primarily an equine disease, it also affects humans^14^ and is a public health concern due to its past use as a bioterrorism agent^15,16^. Beyond melioidosis and glanders, other members of the *Burkholderia* genus also cause severe diseases in humans. The *Burkholderia cepacia* complex comprises of many species within the *Burkholderia* genus that cause a rapid degradation of pulmonary function leading to high mortality rates in cystic fibrosis patients^17–19^. *Burkholderia* species also encode multiple forms of antimicrobial resistance mechanisms^20–22^ further complicating treatment of these diseases and highlighting the need for increased investment in basic research of these organisms at the genetic level.

Among all forms of useful genetic manipulations, techniques have been developed for the rapid generation of chromosomal deletion mutants^23–25^. Although these techniques are less cumbersome than traditional allelic-replacement strategies, they rely on the natural transformative properties of the background strain, limiting the utility of these methods^23–25^. To our knowledge, there are no naturally competent strains of *B*. *mallei* or within the *B*. *cepacia* complex yet described. Thus, the ability to make these *Burkholderia* species uptake DNA would be very significant in manipulating their genomes. Some *Bp* strains are naturally transformable (i.e., ~50% of *Bp* strains)^5,24^. *Bp* strain 1026b is able to naturally uptake extracellular DNA allowing for easy genetic manipulation^23–25^. Prototype strains *Bp* K96243, *Bm* ATCC23344 and *B*. *cenocepacia* (*Bc*) K56-2 are commonly used in the field but are non-naturally competent making genome manipulation tedious and requiring many steps. To further investigate the natural transformation mechanisms of some *Bp* strains and increase the utility of natural transformation-based genetic manipulation techniques, our lab has sought to identify specific genetic properties that confer this phenotype^5^.

Four fosmids containing genomic regions from naturally competent *Bp* 1026b were previously isolated and able to confer natural transformation to non-naturally competent *Burkholderia* species^5^. The fosmids isolated encode ~30–40 genes each, which allowed the non-naturally competent *Bp* K96243, *Bm* ATCC23344 and *Bc* K56-2 to uptake *gfp* DNA and grow on DNA as a sole carbon source^5^. A bioinformatics analysis of each genomic region revealed several candidate genes for natural competency^5^. Mutation in five of these genes, in the naturally competent *Bp* 1026b background, led to a reduction in growth on DNA and *gfp* DNA uptake indicating their involvement in natural transformation^5^. In the present work, we pursued further characterization of the genetic regions of these fosmids and identified the minimal components necessary for natural competency for the purpose of creating a possible broad-species-range strategy for genome manipulation. Additionally, we exploited these genetic elements to expand the λ-Red-recombineering system for rapid chromosomal manipulation into non-naturally competent *Burkholderia* species.

## Results

### Downsized fosmids identify genes responsible for *Burkholderia* natural competency

We initially planned to digest each fosmid (~50 Kbp^5^,), maintained in non-competent *Bp* K96243 or *Bc* K56-2, into smaller genetic fragments in order to identify the minimal number of genes necessary for natural competency. However, working with 50 Kbp inserts on fosmids to pinpoint a subset of genes responsible for natural competency is an arduous task. Therefore, we reintroduced and passaged the fosmid clones in the *Bc* K56-2 background, while maintaining selective pressure for DNA utilization, in anticipation that natural downsizing of the fosmid clones would occur. Upon digestion of the fosmids Bp1 and Bc17^5^, after passage and re-transformation into *Escherichia coli*, it was discovered that each fosmid from *E*. *coli* had been significantly reduced in size (data not shown). The downsized fosmids were re-introduced into *Bc* K56-2 and growth on DNA was confirmed. The downsized fosmids Bp1 and Bc17 contained two open reading frames, BP1026B_I0804 and BP1026B_II2056, respectively. Interestingly, BP1026B_I0804 is highly similar to the known competence protein ComE, which has a high amino acid similarity (56%) and identity (38%) to ComEA of *Neisseria meningitidis*, a model organism for natural competency. Mutation in BP1026B_I0804 (*comE*) significantly reduced the ability of naturally competent *Bp* 1026b to grow on DNA as a sole carbon source and to uptake exogenous DNA^5^. BP1026B_II2056 (*crp*) is a putative transcriptional regulator of the Crp/Fnr family that also showed critical involvement in DNA uptake and catabolism in *Bp* 1026b^5^. Looking at the available genomes of *Bp*, both *comE* and *crp* of *Bp* 1026b exist in 99 and 198 available *Bp* genomes, respectively, even non-naturally competent strains. *Bm* ATCC23344 has *comE* and *crp* homologs to *Bp* 1026b at 100% identity while *Bc* K56-2 has homologs with 66.67% and 73.98% identity, respectively. Moving forward, the natural downsizing event of these fosmids, down to *comE* and *crp*, led us to further investigate the possible transfer of this heritable trait in other *Burkholderia* species.

### *comE* and *crp* allow DNA uptake, utilization, and expression

**Figure 1:**
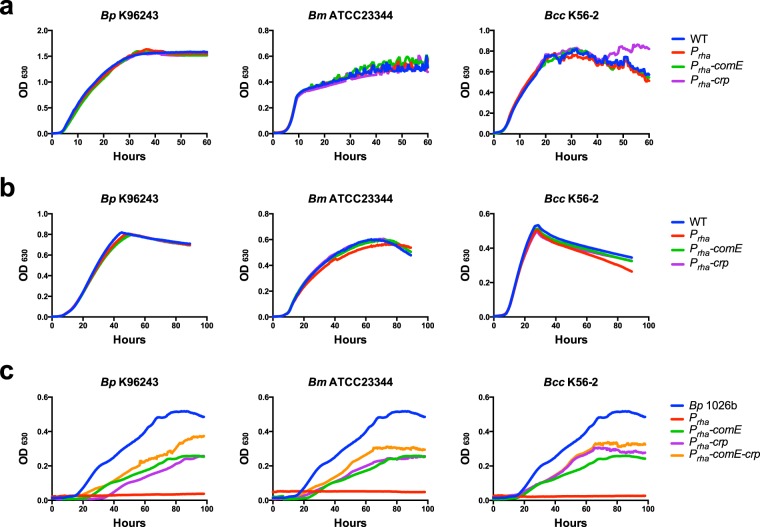
*In vitro* growth characteristics of *Bp* K96243, *Bm* ATCC23344 and *Bc* K56-2. Site-specific recombination at the *attTn7* site was used to insert *comE*, *crp*, or *comE-crp* driven by the rhamnose-inducible promoter (*P*_*rha*_). All strains were tested at 37 °C while shaking in LB **(a)**, M9 minimal media supplemented with 20 mM glucose **(b)**, or minimal media supplemented with 0.1% purified salmon sperm DNA **(c)**. All media contained 0.2% rhamnose to express genes inserted in the *attTn7* site. The naturally competent *Bp* 1026b is shown as a point of reference for growth on DNA as a sole carbon source **(c)**.

To further investigate the roles of *comE* and *crp* in *Burkholderia* natural transformation and competency, we constructed strains that conditionally express each of these proteins, individually and in combination, under the control of a rhamnose-inducible promoter. To reduce the chance that the introduced version of *comE*, *crp*, or *comE-crp* recombine with the native copy of *comE* or *crp*, we exchanged codons throughout each gene to ensure that the nucleotide sequence differed significantly while the amino acid sequence remained unchanged (Supplementary Fig. S1). These combinations were inserted into the *attTn7* site in a diverse group of non-naturally competent pathogenic *Burkholderia* species including *Bp* K96243, *Bm* ATCC2344, and *Bc* K56-2^26,27^. No growth differences were observed when these engineered strains were tested in LB or M9 minimal glucose (MG) media containing rhamnose (Fig. 1a,b). Additionally, empty vector controls (*P*_*rha*_) of non-naturally competent strains *Bp* K96243, *Bm* ATCC23344, and *Bc* K56-2 were unable to grow in media containing DNA as a sole carbon source (red lines in Fig. 1c). However, single copy expression of *comE*, *crp*, or *comE-crp* enabled these non-naturally competent *Burkholderia* strains to grow in DNA to various degrees (Fig. 1c). Although the expression of *comE*, *crp*, or *comE-crp* in these strains did not allow growth to similar levels as the naturally competent *Bp* 1026b, they afforded these non-naturally competent *Burkholderia* strains the ability to significantly uptake DNA as a carbon source and sustain observable growth.

**Figure 2:**
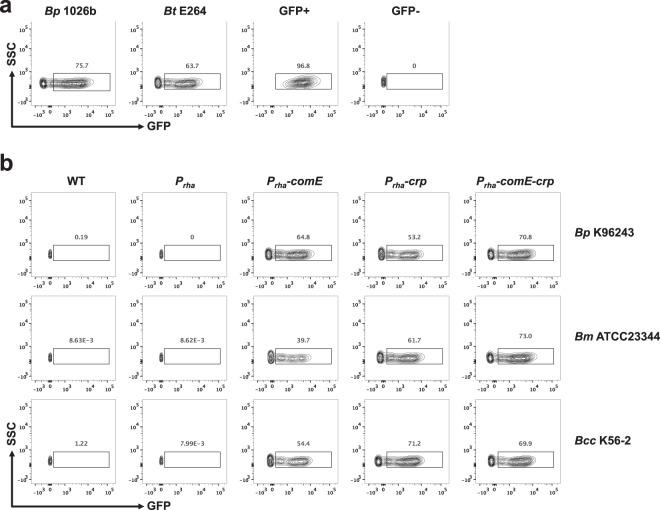
Linear *gfp* DNA uptake assay of strains expressing *comE*, *crp*, or *comE-crp*. For all plots side scatter (SSC) is plotted against GFP fluorescent intensity (GFP). **(a)** Naturally competent *Bp* 1026b and *Bt* E264 show 63–75% of cells expressing GFP after incubation with *gfp*. *E*. *coli* constitutively expressing *gfp* (GFP+) shows 96.8% of cells expressing GFP in contrast to wildtype *E*. *coli* (GFP-) showing no GFP expression. **(b)** Wildtype (WT) and *attTn7* controls (*P*_*rha*_) of *Bp* K96243, *Bm* ATCC23344 and *Bc* K56-2 show no GFP expression indicating their inability to uptake *gfp*. However, expression of *comE*, *crp* or *comE-crp* empowered natural competency, showing 39–73% of cells expressing GFP.

Beyond DNA catabolism, we tested DNA uptake and expression characteristics provided by *comE*, *crp*, or *comE-crp* to these non-naturally competent *Burkholderia* species (Fig. 2). Each engineered strain was incubated with linear *gfp* DNA^24^ and the level of GFP uptake and transient expression of GFP was quantitated using flow cytometry. To establish GFP detection parameters, an *E*. *coli* strain constitutively expressing GFP was compared to an *E*. *coli* strain with no GFP expression. The *E*. *coli* strain constitutively expressing GFP showed 96.8% of cells GFP+, while the *E*. *coli* strain that is *gfp-*, showed no GFP expression (Fig. 2a). When incubated with *gfp* DNA, 75.7% and 63.7% of naturally competent *Bp* 1026b and *Burkholderia thailandensis* (*Bt*) E264 were able to uptake and express GFP, respectively (Fig. 2a). Non-naturally competent *Bp* K96243, *Bm* ATCC23344, and *Bc* K56-2 strains displayed no GFP expression (Fig. 2b, WT and *P*_*rha*_ columns). In contrast, these non-naturally competent strains that express *comE*, *crp*, or *comE-crp* showed GFP expression ranging from 39.7% to 73%, demonstrating that *comE* or *crp* is able to confer DNA uptake and subsequent expression of exogenous DNA (Fig. 2b). The capability of DNA uptake enabled by *comE* and *crp* made them promising candidates for testing in conjunction with genetic manipulation techniques that rely on natural transformation in non-naturally competent strains. In the presence of *comE-crp*, the high frequencies of cells taking up *gfp* DNA by *Bc* K56-2 (69.9% of cells) and *Bm* ATCC23344 (73% of cells) were comparable to wildtype *Bp* strain 1026b (75.7% of cells) and *Bt* strain E264 (63.7% of cells, Fig. 2). Because manipulating and modifying the genomes of *Bp* 1026b and *Bt* E264 has been highly dependent upon the ability to uptake DNA by natural competency^24,25^ the high frequencies of this heritable trait (Fig. 2) indicate that genetic manipulation of the genome of *Bm* and *Bc* is very much possible. As proof-of-concept, we next utilized *comE-crp* to manipulate the genome of four different *Bm* strains. A codon-altered version of *comE-crp* was synthesized to prevent recombination between *comE-crp* on plasmid and the native genomic *comE* and *crp* copies.

### Genetic manipulation of non-naturally transformable *Burkholderia spp*

**Figure 3:**
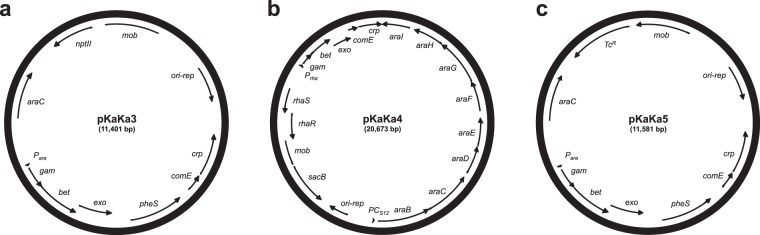
Plasmid maps of pKaKa3, pKaKa4, and pKaKa5 to expand the utility of λ-red recombineering to non-naturally competent *Burkholderia* species. Abbreviations: *araC* on pKaKa3 and pKaKa5, activator of the arabinose-inducible promoter (P_*ara*_) from *E*. *coli*; *araBCDEFGHI* on pKaKa4, *B*. *thailandensis* arabinose utilization operon^38^; *gam*-*exo*-*bet*, λ-red recombineering genes^39^; *mob*; RP4-dependent conjugal origin of transfer of *B*. *bronchiseptica* cryptic plasmid pBBR1; *ori-rep*; bhr replicon of *B*. *bronchiseptica* pBBR1 plasmid^40^; *nptII*, encodes kanamycin resistance^41^; P_*ara*_, arabinose inducible promoter^42^; P_*rha*_, rhamnose inducible promoter^43^; PC_*S12*_, constitutive promoters of *B*. *pseudomallei* and *B*. *cenocepacia rpsL* gene^44^; *pheS*, engineered gene encoding a mutant version of α-subunit of phenylalanyl tRNA synthase^45^; *rhaR* and *rhaS*, regulators of the rhamnose inducible promoter^43^; *sacB*, encoding for a modified levansucrase counter-selectable marker^46^. Tc^R^, tetracycline resistance.

To expand the use of genetic manipulation techniques that rely on natural transformation^23–25^, we created pKaKa3, pKaKa4, and pKaKa5 where each only differ in the resistance marker (Fig. 3). These vectors expand the applicability of the λ-red recombineering system^24^ to non-naturally competent *Burkholderia* species, including type strains *Bp* K96243, *Bm* ATCC23344, and *Bc* K56-2. Incorporation of the codon-altered version of *comE-crp* into the λ-Red-recombineering system will allow DNA uptake and rapid generation of mutants in strains that are non-naturally competent. As proof of concept, we tested this newly designed recombineering system in the non-competent select-agent *Bm* using pKaKa4. The gene encoding aspartate-semialdehyde-dehydrogenase (*asd*) was targeted to generate potentially attenuated strains that could also be useful to the research field^28^. The vector pKaKa4 was introduced into a variety of *Bm* strains (ATCC23344, Ivan, China 5, and 2002721278), and the chromosomal *asd* gene was deleted by incubating with a DNA fragment containing a *gat-pheS-FRT* cassette flanked by 45 bp regions homologous to *Bm asd*. Glyphosate-resistant colonies of *Bm* strains were purified and their diaminopimelate (DAP) requiring phenotype verified (data not shown). The pKaKa4 plasmid with the *sacB* gene was cured by counter-selection on sucrose. Recombinant efficiencies varied among different *Bm* strains but generally, 10–50 colonies were obtained from a typical experiment when approximately 5 × 10^8^ to 1 × 10^9^ CFU were used. The introduction of *comE-crp* enabled *Bm* to uptake and recombine DNA, which was previously impossible. Although the focus of the present study was to conditionally attenuated *Bm* strains, we have also utilized these genetic tools successfully in non-naturally competent *Bc* K56–2 and *Bp* K96243 to manipulate their genomes with similar frequencies of recombinants.

### Attenuation of *Bm* Δ*asd* mutants in intracellular replication and acute glanders models

**Figure 4:**
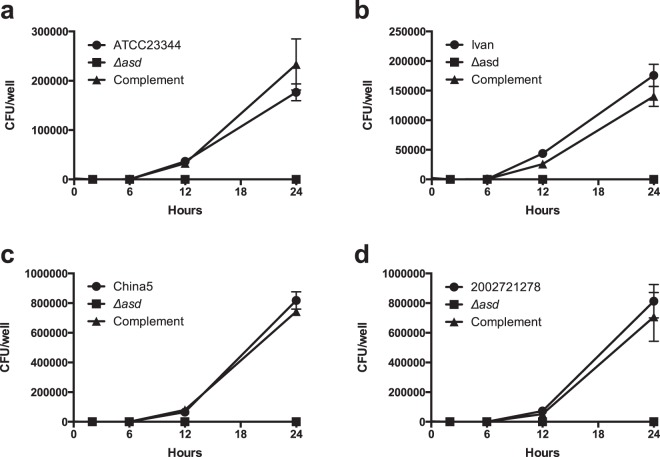
*In vitro* attenuation of *Bm* Δ*asd* strains in RAW264.7 murine macrophages. *Bm* strains ATCC23344 **(a)**, Ivan **(b)**, China 5 **(c)**, 2002721278 **(d)** were able to replicate well within the intracellular environment (circles) while the Δ*asd* counterparts showed complete abolishment of the ability to replicate intracellularly (squares). Complementation of the Δ*asd* gene in each *Bm* strain rescued the intracellular replication defect (triangles).

To determine the level of attenuation of the four *Bm* Δ*asd* strains produced using the natural transformation properties of *comE-crp*, we first tested them in a RAW264.7 murine macrophage model of infection^28^. RAW264.7 cells were infected with wildtype *Bm* ATCC23344, Ivan, China5, 2002721278 and the Δ*asd* mutants of each, at an MOI of 1:1 in a modified kanamycin protection assay in order to assess each mutants ability to infect intracellularly. Wildtype *Bm* strains ATCC23344 (Fig. 4a), Ivan (Fig. 4b), China 5 (Fig. 4c), and 2002721278 (Fig. 4d) were able to replicate to high levels intracellularly, while all *Bm* Δ*asd* strains behaved as expected, showing no replication within the intracellular environment where no DAP was present. Single-copy complementation of each *Bm* Δ*asd* strain recovered this defect, indicating that the defect in intracellular replication was due to the deletion of the *asd* gene (Fig. 4, triangles).

**Figure 5:**
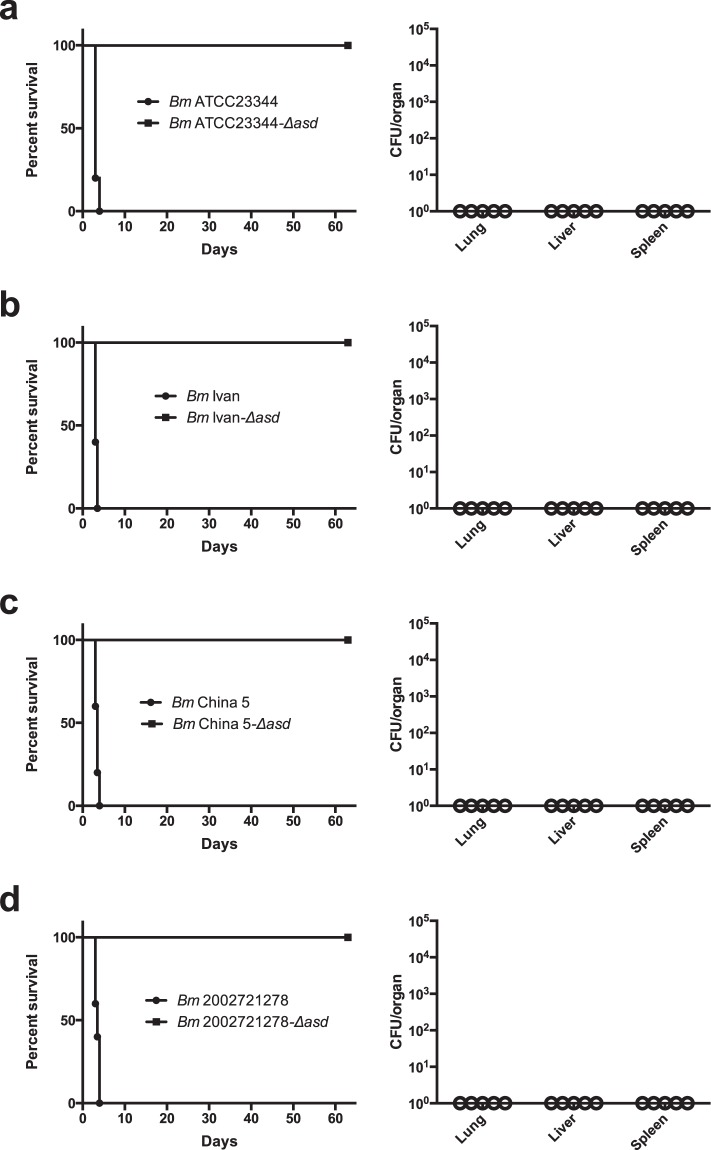
*In vivo* attenuation of *Bm* Δ*asd* strains in BALB/c intranasal challenge. BALB/c mice (n = 5) were challenged intranasally with 1 × 10^7^ CFU of *Bm* strains ATCC23344 **(a)**, Ivan **(b)**, China 5 **(c)**, 2002721278 **(d)** and their Δ*asd* counterparts. Survival was monitored for 63 days (left panels). Surviving mice were sacrificed and bacterial burdens from the lungs, liver, and spleen were determined by serial dilution and plating (right panels).

In addition to *in vitro* attenuation, we sought to test the *Bm* Δ*asd* strains in an acute glanders model. To best mimic inhalation glanders, we infected BALB/c mice via intranasal inoculation with an intentionally high-dose of each strain that leads to acute pneumonic glanders. Groups of five mice were inoculated with 1 × 10^7^ CFU of wildtype *Bm* strain or its Δ*asd* counterpart and survival was monitored. Mice infected with wildtype *Bm* strains ATCC23344 (Fig. 5a), Ivan (Fig. 5b), China 5 (Fig. 5c), and 2002721278 (Fig. 5d) rapidly deteriorated showing severe symptoms of acute glanders and had to be euthanized within the first four days of the trial. In contrast, BALB/c mice inoculated with *Bm* Δ*asd* strains showed no signs or symptoms of disease and survived until the study was terminated at day 63 (Fig. 5, squares). Bacterial burdens from the lungs, liver, and spleen were assessed in surviving mice to determine any level of *Bm* Δ*asd* mutant persistence within the host. Organs were homogenized and plated onto LB agar containing DAP. *Bm* Δ*asd* were not detected in any organ, indicating that the mutant strains were not able to persist within the host (Fig. 5).

## Discussion

Natural transformation is a complex process that drives genetic diversification, DNA repair, and DNA catabolism in bacteria^2^. The amount of bacterial species identified as naturally transformable is increasing as the molecular mechanisms that drive this activity are better understood in model organisms^3^. Although the exact mechanism and relationship between DNA up-take and catabolism is yet to be determined, we summarized as previously depicted that one strand of the double-stranded DNA is broken down for catabolism and the other strand can enters the cell for transformation^5^. Only some strains of *Bp* are naturally competent^5,24^, a phenomenon that is not unique to *Bp*, but also found in the emerging plant pathogen *Xylella fastidiosa*^29^. The mechanism by which certain strains of *Bp* are naturally competent is not well understood. Crp has been implicated as a regulatory factor in the process of natural competency in many organisms including *Vibrio cholerae*^30^ and *Haemophilus influenzae*^31^. We therefore hypothesize that Crp in *Burkholderia* species plays a critical role in the regulation of competence, supported by the data presented here. The regulation network of Crp in *Burkholderia* is of critical interest for future studies and could reveal valuable insights into the complex mechanism of natural competency. ComE has been known to be involved in natural competency of many gram-negative organisms^3^ including *N*. *gonorrhoeae* and *N*. *meningitides*, which have shown a direct correlation with the copy number of *comE* and the level of competency^32^. This supports the conclusion that an additional copy of *comE* expressed in non-naturally competent *Burkholderia* would lead to increase levels of DNA uptake and catabolism above the threshold of detection. Although, no significant additive affect was observed when *comE* and *crp* were introduced in combination to the non-naturally competent backgrounds, our data did indicate that overall the *comE*-*crp* gave rise to a higher ability for DNA catabolism and uptake, compared to *comE* or *crp* individually (Figs 1 and 2).

The fosmids previously isolated^5^ narrowed down the genetic elements for DNA uptake and utilization to ~50Kbp. The fosmids themselves were naturally downsized further during selection in *Burkholderia* and maintenance in *E*. *coli*, leading us to investigate the role of the remaining genetic elements, *comE* and *crp*. Individually and in combination, *comE* and *crp* conferred the ability for non-naturally competent *Bp* K96243 to grow on DNA as a sole carbon source. In addition to DNA catabolism, the ability to uptake and express *gfp* DNA was also observed, solidifying the role that *comE* and *crp* play in *Burkholderia* natural transformation. To broaden the scope of these findings, we investigated a diverse range of non-naturally competent *Burkholderia* species that are of public health concern. These include the closely related but distinct *Bm*, the etiological agent of glanders, and the more distantly related *Bc*, one of the agents that cause the cepacia syndrome in cystic fibrosis patients. The expression of *comE* and *crp* in both *Bm* ATCC23344 and *Bc* K56-2 conferred the ability to catabolize DNA and uptake and express exogenous *gfp* DNA, indicating that the competence machinery in *Burkholderia* species is likely similar.

Researchers have gravitated toward utilizing *Bp* 1026b and *Bt* E264 because these are naturally competent and, therefore, easier to manipulate genetically. However, *Bp* K96243 was one of the first *Bp* genomes sequenced and is a prototype strain. The genetic manipulation of *Bp* K96243 has been limited due to its inability to uptake DNA efficiently. Likewise, the genetic manipulation of *Bm* strains and *Bc* K56-2 has been tedious because of the inefficiency in DNA uptake. We showed here *Bp* K96243, *Bc* K56-2, and *Bm* strains can inherit the high frequencies of *gfp* DNA uptake comparable to wildtype *Bp* 1026b and *Bt* E264 (Fig. 2), alleviating the difficulty in manipulating the genomes of these bacteria to knock-out and pull-out genomic sequences^24^.

In the present study, we also developed genetic tools to expand the λ-Red recombineering system to include non-naturally competent *Burkholderia* species and strains. Three different λ-Red recombineering vectors were constructed based on various antibiotic and non-antibiotic selective markers, as well as counter-selective markers for curing of the vectors, making them broad-host-range. As a proof of concept, pKaKa4 was used successfully to mutate the *asd* gene from four strains of *Bm*. These mutants show complete attenuation in cell culture and BALB/c models of infection and a request for the exclusion from the select-agent list has been submitted and approved by the CDC (https://www.selectagents.gov/SelectAgentsandToxinsExclusions.html). The exclusion of these *Bm* strains will help accelerate the study of glanders and could be of great interest to the research field. Furthermore, development of these novel genetic tools significantly simplifies the genetic manipulation in many other non-naturally competent *Burkholderia* species/strains, allowing high-throughput targeted chromosomal manipulation.

## Methods and Materials

### Bacterial strains, media and culture conditions

All manipulation of *Bp* and *Bm* were conducted in a CDC-approved and -registered BSL3 facility at the University of Hawaii at Manoa (UHM). All select agent experiments were approved by the Institutional Biosafety Committee of UHM (reference number: 16–07–004–585–1 R) and were performed using BSL3 practices following recommendations set forth in the BMBL, 5^th^ edition^33^. *Escherichia coli* strain EPMax10B (BioRad), E1869, and E1354 were routinely used for cloning or plasmid mobilization into *Bp*, *Bm* and *Bc* as described previously^27,34^. Luria-Bertani (LB) medium (Difco) or 1x M9 minimal medium supplemented with 20 mM glucose (MG) or 0.1% salmon sperm DNA was used to culture all strains. Induction of genes controlled by the rhamnose-inducible promoter (*P*_*rha*_) was done as previously described^24^. Selection of the *gat* gene in *E*. *coli* and *Bp* strains was performed as previously described^34^. Fosmid downsizing: fosmids^5^ were isolated from *E*. *coli* and introduced into *Bc* K56–2 and selected on plates with DNA as a sole carbon source. Colonies of *Bc* K56–2 containing the fosmids from DNA plates were grown up in liquid media with DNA, fosmids were re-purified from the liquid cultures and re-transformed into *E*. *coli* and tested for a downsizing event. Downsized fosmids were tested for growth on DNA in *Bc* K56-2 as previously described^5^.

### Molecular methods and reagents

Molecular methods and reagents were carried out as described previously^24,28,34,35^. Versions of both *comE* and *crp* genes were designed to avoid recombination between the introduced copy of *comE-crp* and the native genomic *comE* and *crp*. To achieve this, codons were swapped throughout each gene to change the nucleotide sequence without altering the amino acid sequence (Supplementary Fig. S1). Newly designed *comE* and *crp* genes were synthesized through Genscript^®^. Strains conditionally expressing *comE*, *crp*, and *comE-crp* were constructed utilizing mini-Tn7 integration vectors^26,27,34^. Briefly, the rhamnose inducible promoter fragment was PCR amplified from pFlpe4^23^ using oligos 5′-CATATGCATTTAATCTTTCTGCGA-3′ and 5′-CGACTAGTGGATATCGAACTGGCTCATG-3′, digested with *Nsi*I and *Spe*I, and cloned into mini-Tn7-*gat*^34^ digested with the same enzymes, yielding mini-Tn7-*gat-*P*rha*. Newly synthesized *comE*, *crp*, *and comE-crp* were cloned into mini-Tn7-*gat-Prha* as *Bam*HI/*Hin*dIII, *Hin*dIII/*Spe*I-blunted, and *Bam*HI/*Spe*I-blunted fragments, respectively. These plasmids were conjugated into non-naturally competent *Burkholderia* strains and insertion into the *attTn7* site was screened as previously described^26,27^.

### Growth analysis of *Burkholderia* species

All strains were first grown overnight in LB at 37 °C, bacteria were harvested and washed twice with 1xM9 minimal media and subcultured 1:200 into fresh LB, M9 minimal media supplemented with 20 mM glucose, or minimal media supplemented with 0.1% purified salmon sperm DNA (Fig. 1). All media contained 0.2% rhamnose to express genes inserted in the *attTn7* site. Growth curves were done using the BioTek ELx808IU by measuring OD_630_ every 30 minutes for the duration of the time course. Growth analysis was done in triplicate and average ODs were shown.

### *Gfp* uptake assays

*Gfp* uptake assays were performed as previously described^5,24^, with the exception of the detection method. Briefly, *gfp*-DNA was amplified by PCR from pPS747^36^ and 250 ng of the *gfp*-DNA was incubated with various strains for 30 min at room temperature^5^. After 45 min recovery in LB broth with shaking, bacteria were fixed in 1% paraformaldehyde in 1x phosphate buffered saline (PBS) for 45 min for fluorescent analysis. After fixation, bacteria were harvested and resuspended gently with 1xPBS + 0.1% Triton X-100 to reduce clumping, and then washed twice with 1xPBS to remove detergent. Fixed bacteria were analyzed using flow cytometry to detect transient expression of GFP-protein indicating that cells were able to uptake extracellular *gfp-*DNA, along with fixed *E*. *coli* wildtype strain DH5α and DH5α/*attB*::Gm-*gfp* as negative and positive controls, respectively.

### λ-Red knockout recombineering in *Bm* with pKaKa4

Generation of mutants was done as previously described^24^ with slight modifications. Briefly, pKaKa4 was introduced into various *Bm* strains via conjugation and selection on M9 minimal media containing 40 mM arabinose as the sole carbon source. *Bm* strains harboring pKaKa4 were streaked out on M9 + arabinose plates and grown for 3 days at 37 °C, then harvested from plates by gentle scraping and resuspended in fresh LB containing 0.2% rhamnose. Bacteria were then concentrated by centrifugation and resuspended in 20 μl LB + 0.2% rhamnose, and incubated with 2 μg of DNA containing a *gat-pheS-FRT* cassette flanked by 45 bp regions homologous to *Bm asd*. After incubation at room temperature for 30 min, bacteria were recovered in fresh LB for 2 hours at 37 °C, and selected on MG medium containing 200 μg/ml DAP, 0.4% GS, and 1 mM each of lysine, methionine, and threonine (these 3 amino acids are required for the specific *asd* mutation).

### Intracellular replication assays

RAW264.7 murine macrophages were grown in Dulbecco’s modified Eagle’s medium (DMEM) supplemented with 10% (v/v) fetal bovine serum (FBS) at 37 °C in 5% CO_2_. Antibiotic/antimycotic (Gibco) containing 100 U/mL penicillin, 100 µg/mL streptomycin and 250 ng/mL of amphotericin B was added to media at a 1X concentration during cell growth but omitted during infection trials. A modified kanamycin protection assay was used test intracellular replication^37^. RAW264.7 cells were seeded into 24-well Corning CellBIND culture plates to 80% confluence, allowed to attach overnight, and were washed twice with 1XPBS before infection. *Bm* Δ*asd* strains were used to infect macrophage monolayers at an MOI of 1:1. After 1 hour, infected monolayers were washed with 1XPBS and then DMEM supplemented with 10% FBS, 700 µg/mL amikacin and 700 µg/mL kanamycin were added to kill any extracellular bacteria. At 2, 6, 12, and 24 hours post-infection, infected monolayers were lysed with 0.1% Triton X-100. Serial dilutions of lysates were plated on LB containing 200 µg/mL DAP and colony forming units (CFU) per well were determined.

### Animal studies

BALB/c mice between 4 and 6 weeks of age were purchased from Charles River Laboratory. All infections with *Bm* strains were administered via the intranasal (i.n.) inoculation route. Mice were anesthetized with 100 mg of ketamine/kg of body weight plus 10 mg/kg xylazine. The challenge dose (1 × 10^7^ CFU) of each *Bm* strain was suspended in 20 µl of 1XPBS and used to inoculate each mouse via the i.n. route. Each strain was used to inoculate 5 mice. Animals were monitored for disease symptoms daily and euthanized at predetermined humane end points. Lungs, liver, and spleen of surviving mice were harvested, homogenized, serially diluted, and plated on LB containing 200 µg/mL DAP to determine bacterial burdens. Survival characteristics were plotted using Prism software (GraphPad, La Jolla, CA) and statistical analysis was done by Kaplan-Meier curves.

### Ethics statement

All animal studies described in this manuscript were approved by the Institutional Animal Care and Use Committee at the University of Hawaii at Manoa (Protocol No. 10-1073-8), and conducted in compliance with the NIH (National Institutes of Health) Guide for the Care and Use of Laboratory Animals.

## Electronic supplementary material

Supplementary information

## Data Availability

The datasets and materials generated during the current study are available from the corresponding author upon reasonable request. Any transfer of select agent materials must be to a select agent registered facility, approved by the CDC, and comply with all select agent regulations (selectagents.gov).

## References

[1] Griffith F (1928). The Significance of Pneumococcal Types. J Hyg (Lond).

[2] Chen I, Dubnau D (2004). DNA uptake during bacterial transformation. Nat Rev Microbiol.

[3] Johnston C, Martin B, Fichant G, Polard P, Claverys JP (2014). Bacterial transformation: distribution, shared mechanisms and divergent control. Nat Rev Microbiol.

[4] Aas FE (2002). Competence for natural transformation in Neisseria gonorrhoeae: components of DNA binding and uptake linked to type IV pilus expression. Mol Microbiol.

[5] Norris MH (2017). Burkholderia pseudomallei natural competency and DNA catabolism: Identification and characterization of relevant genes from a constructed fosmid library. PLoS One.

[6] Croucher NJ (2011). Rapid pneumococcal evolution in response to clinical interventions. Science.

[7] Cheng AC, Currie BJ (2005). Melioidosis: epidemiology, pathophysiology, and management. Clinical Microbiological Reviews.

[8] Limmathurotsakul, D. *et al*. Predicted global distribution of and burden of melioidosis. *Nat Microbiol* **1** (2016). 10.1038/nmicrobiol.2015.8 27571754

[9] Nandi T (2015). Burkholderia pseudomallei sequencing identifies genomic clades with distinct recombination, accessory, and epigenetic profiles. Genome Res.

[10] McRobb E (2014). Distribution of Burkholderia pseudomallei in northern Australia, a land of diversity. Appl Environ Microbiol.

[11] Cheng AC (2008). Genetic diversity of Burkholderia pseudomallei isolates in Australia. J Clin Microbiol.

[12] Price, E. P. *et al*. Within-host evolution of Burkholderia pseudomallei over a twelve-year chronic carriage infection. *MBio* **4** (2013). 10.1128/mBio.00388-13 PMC3735121 23860767

[13] Losada L (2010). Continuing evolution of Burkholderia mallei through genome reduction and large-scale rearrangements. Genome Biol Evol.

[14] Srinivasan A (2001). Glanders in a military research microbiologist. N Engl J Med.

[15] Larsen JC, Johnson NH (2009). Pathogenesis of Burkholderia pseudomallei and Burkholderia mallei. Mil Med.

[16] Frischknecht F (2003). The history of biological warfare. Eur. Mol. Biol. Org..

[17] Isles A (1984). Pseudomonas cepacia infection in cystic fibrosis: an emerging problem. J Pediatr.

[18] Corey M (1996). & Farewell, V. Determinants of mortality from cystic fibrosis in Canada, 1970-1989. Am J Epidemiol.

[19] Mahenthiralingam E, Urban TA, Goldberg JB (2005). The multifarious, multireplicon Burkholderia cepacia complex. Nat Rev Microbiol.

[20] Podnecky NL, Rhodes KA, Schweizer HP (2015). E ffl ux pump-mediated drug resistance in Burkholderia. Front Microbiol.

[21] Rholl DA (2011). Molecular Investigations of PenA-mediated beta-lactam Resistance in Burkholderia pseudomallei. Front Microbiol.

[22] Chantratita N (2011). Antimicrobial resistance to ceftazidime involving loss of penicillin-binding protein 3 in Burkholderia pseudomallei. Proc Natl Acad Sci USA.

[23] Choi KH (2008). Genetic tools for select-agent-compliant manipulation of *Burkholderia pseudomallei*. Appl. Environ. Microbiol..

[24] Kang Y (2011). Knock-out and pull-out recombinant engineering protocols for naturally transformable *Burkholderia thailandensis* and *Burkholderia pseudomallei*. Nature Protocols.

[25] Thongdee M (2008). Targetted mutagenesis of *Burkholderia pseudomallei* and *Burkholderia thailandensis* through natural transformation of PCR fragments. Appl. Environ. Microbiol..

[26] Choi KH, DeShazer D, Schweizer HP (2006). mini-Tn7 insertion in bacteria with multiple glmS-linked attTn7 sites: example Burkholderia mallei ATCC 23344. Nat Protoc.

[27] Kang Y, Norris MH, Barrett AR, Wilcox BA, Hoang TT (2009). Engineering of tellurite-resistant genetic tools for single-copy chromosomal analysis of Burkholderia spp. and characterization of the Burkholderia thailandensis betBA operon. Appl Environ Microbiol.

[28] Norris MH (2011). The Burkholderia pseudomallei Deltaasd mutant exhibits attenuated intracellular infectivity and imparts protection against acute inhalation melioidosis in mice. Infect Immun.

[29] Kandel PP, Almeida RPP, Cobine PA, De La Fuente L (2017). Natural Competence Rates Are Variable Among Xylella fastidiosa Strains and Homologous Recombination Occurs *In Vitro* Between Subspecies fastidiosa and multiplex. Mol Plant Microbe Interact.

[30] Blokesch M (2012). Chitin colonization, chitin degradation and chitin-induced natural competence of Vibrio cholerae are subject to catabolite repression. Environ Microbiol.

[31] Redfield RJ (2005). A novel CRP-dependent regulon controls expression of competence genes in Haemophilus influenzae. J Mol Biol.

[32] Chen I, Gotschlich EC (2001). ComE, a competence protein from Neisseria gonorrhoeae with DNA-binding activity. J Bacteriol.

[33] Wilson, D. E. & Chosewood, L. C. Biosafety in microbiological and biomedical laboratories (BMBL), 5th ed. Centers for Disease Control and Prevention, Atlanta, GA. (2007).

[34] Norris MH, Kang Y, Lu D, Wilcox BA, Hoang TT (2009). Glyphosate resistance as a novel select-agent-compliant, non-antibiotic selectable marker in chromosomal mutagenesis of the essential genes *asd* and *dapB* of *Burkholderia pseudomallei*. Appl. Environ. Microbiol..

[35] Norris MH, Kang Y, Wilcox B, Hoang TT (2010). Stable site-specific fluorescent tagging constructs optimized for *Burkholderia* species. Appl Environ Microbiol.

[36] Hoang TT, Karkhoff-Schweizer RR, Kutchma AJ, Schweizer HP (1998). A broad-host-range Flp-*FRT* recombination system for site-specific excision of chromosomally-located DNA sequences: application for isolation of unmarked *Pseudomonas aeruginosa* mutants. Gene.

[37] Jones AL, Beveridge TJ, Woods DE (1996). Intracellular survival of *Burkholderia pseudomallei*. J. Bacteriol..

[38] Moore RA (2004). Contribution of gene loss to the pathogenic evolution of *Burkholderia pseudomallei* and *Burkholderia mallei*. Infect. Immun..

[39] Nakayama M, Ohara O (2005). Improvement of recombination efficiency by mutation of Red proteins. BioTechniques.

[40] Antoine R, Locht C (1991). Isolation and molecular characterization of a novel broad-host-range plasmid from *Bordetella bronchiseptica* with sequence similarities to plasmids from Gram-positive organisms. Mol. Microbiol..

[41] Yu M, Tsang JSH (2006). Use of ribosomal promoters from *Burkholderia cenocepacia* and *Burkholderia cepacia* for improved expression of transporter protein in *Escherichia coli*. Protein Expr. Purif..

[42] Datsenko KA, Wanner BL (2000). One-step inactivation of chromosomal genes in *Escherichia coli* K-12 using PCR products. Proc. Natl. Acad. Sci. USA.

[43] Baba, T. *et al*. Construction of Escherichia coli K-12 in-frame, single-gene knockout mutants: the Keio collection. *Mol Syst Biol* **2** (2006). 10.1038/msb4100050 PMC1681482 16738554

[44] DeShazer D, Brett PJ, Carlyon R, Woods DE (1997). Mutagenesis of *Burkholderia pseudomallei* with Tn5-OT182: isolation of motility mutant and molecular characterization of the flagellin structural gene. J. Bacteriol..

[45] Barrett AR (2008). Genetic tools for allelic replacement in *Burkholderia* species. Appl. Environ. Microbiol..

[46] Lopez CM, Rholl DA, Trunck LA, Schweizer HP (2009). Versatile Dual-Technology System for Markerless Allele Replacement in Burkholderia pseudomallei. Appl. Environ. Microbiol..

