# Supplementary material for: The heritable natural competency trait of Burkholderia pseudomallei in other Burkholderia species through comE and crp
Source: Sci Rep. 2018 Aug 20;8:12422. doi: 10.1038/s41598-018-30853-4 (PMC6102250; doi:10.1038/s41598-018-30853-4)
Supplement: Supplementary file 1 — Supplementary information [file 41598_2018_30853_MOESM1_ESM.pdf]

The heritable natural competency trait of *Burkholderia pseudomallei* in other *Burkholderia* species through *comE* and *crp*

Yun Heacock-Kang[1], Ian A. McMillan[1,2], Jan Zarzycki-Siek[1], Zhenxin Sun[1], Andrew P. Bluhm[1], Darlene Cabanas[1], and Tung T. Hoang[1,2*]

[1]*Department of Microbiology,* [2]*Department of Molecular Biosciences and Bioengineering, University of Hawaii at Manoa, Honolulu, Hawaii, USA*

* Address correspondence to Tung T. Hoang, Tel: +1 808 956 3522; Fax: +1 808 956 5339; e-mail: tongh@hawaii.edu

Supplementary Figure S1| Nucleotide alignments comparing the native and altered versions of *comE* (a) and *crp* (b) showing the changes in sequence throughout the entire gene to reduce the possibility of recombination. Amino acid alignments comparing native and altered versions of *comE* (c) and *crp* (d) showing 100% identity of the protein sequence. Residues highlighted by red indicate a change in sequence. Sequence conservation is shown by pink bars below the sequence. Alignments were carried out in CLC sequence viewer.

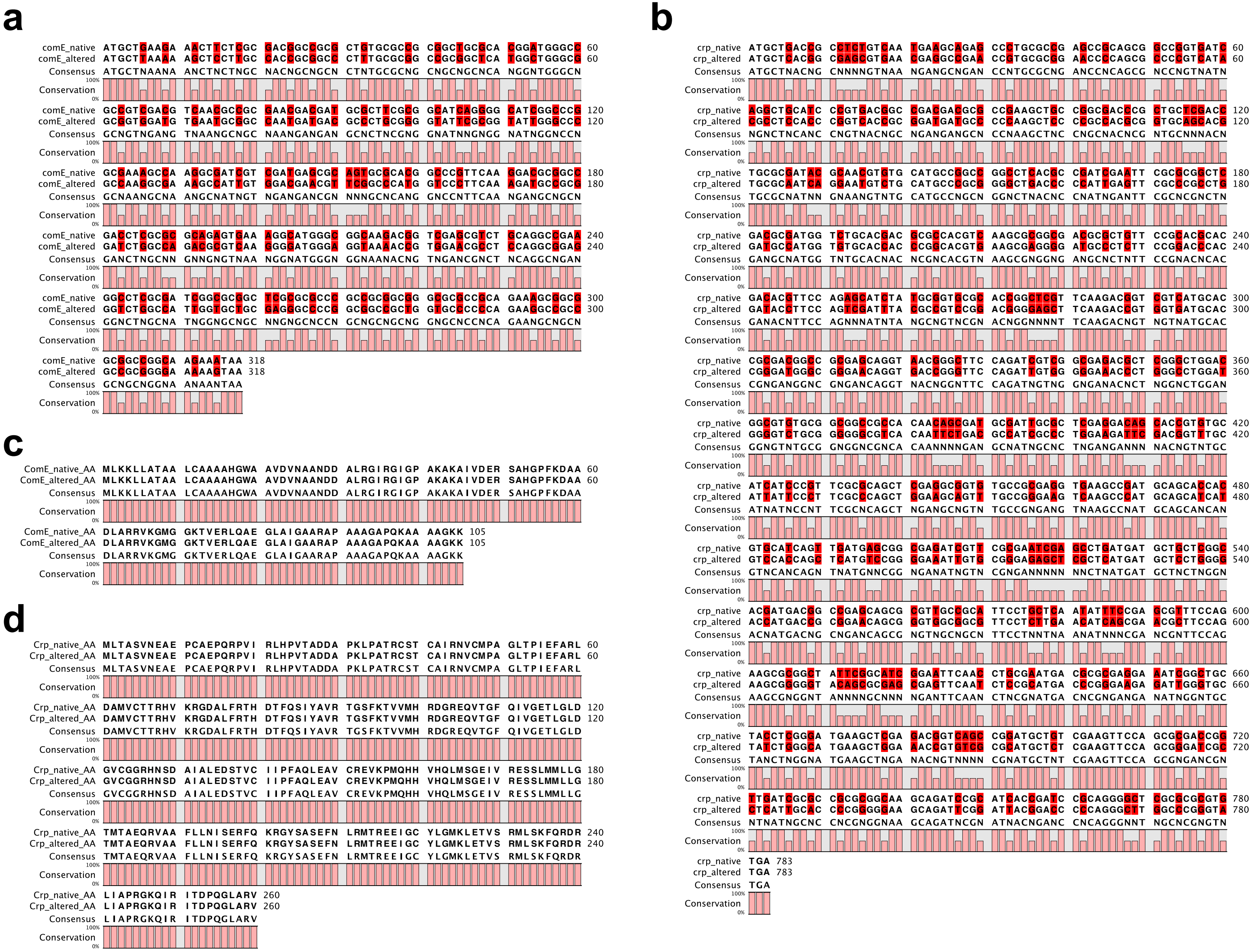
a
comE_native ATGCTGAAGA AACTTCTCGC GACGGCCGCG CTGTGCGCCG CGGCTGCGCA CGGATGGGCC 60
comE_altered ATGCTTAAAA AGCTCCTTGC CACCGCGGCC CTTTGCGCGG CCGCGGCTCA TGGCTGGGCG 60
Consensus ATGCTNAANA ANCTNCTNGC NACNGCNGCN CTNTGCGCNG CNGCNGCNCA NGGNTGGGCN
Conservation
comE_native GCCGTCGACG TCAACGCCGC GAACGACGAT GCGCTTCGCG GCATCAGGGG CATCGGCCCG 120
comE_altered GCGGTGGATG TGAATGCGGC CAATGATGAC GCCCTGCGGG GTATTCGCGG TATTGGCCCC 120
Consensus GCNGTNGANG TNAANGCNGC NAANGANGAN GCNCTNCGNG GNATNNGNGG NATNGGNCCN
Conservation
comE_native GCGAAAGCCA AGGCGATCGT CGATGAGCGC AGTGCGCACG GCCCGTTCAA GGACGCGGCC 180
comE_altered GCCAAGGCGA AAGCCATTGT GGACGAACGT TCGGCCCATG GTCCCTTCAA AGATGCCGCG 180
Consensus GCNAANGCNA ANGCNATNGT NGANGANCGN NNNGCNCANG GNCCNTTCAA NGANGCNGCN
Conservation
comE_native GACCTCGCGC GCAGAGTGAA AGGCATGGGC GGCAAGACGG TCGAGCGTCT GCAGGCCGAA 240
comE_altered GATCTGGCCA GACGCGTCAA GGGGATGGGA GGTAAAACCG TGGAACGCCT CCAGGCGGAG 240
Consensus GANCTNGCNN GNNGNGTNAA NGGNATGGGN GGNAANACNG TNGANCGNCT NCAGGCNGAN
Conservation
comE_native GGCCTCGCGA TCGGCGCGGC TCGCGCGCCC GCCGCGGCGG GCGCGCCGCA GAAAGCGGCG 300
comE_altered GGTCTGGCCA TTGGTGCTGC GAGGGCCCCG GCGGCCGCTG GTGCCCCCCA GAAGGCCGCC 300
Consensus GGNCTNGCNA TNGGNGCNGC NNGNGCNCCN GCNGCNGCNG GNGCNCCNCA GAANGCNGCN
Conservation
comE_native GCGGCCGGCA AGAAATAA 318
comE_altered GCCGCCGGGA AAAAGTAA 318
Consensus GCNGCNGGNA ANAANTAA
Conservation
b
crp_native ATGCTGACCG CCTCTGTCAA TGAAGCAGAG CCCTGCGCCG AGCCGCAGCG GCCGGTGATC 60
crp_altered ATGCTCACGG CGAGCGTGAA CGAGGCCGAA CCGTGCGCGG AACCCCAGCG CCCCGTCATA 60
Consensus ATGCTNACNG CNNNNGTNAA NGANGCNGAN CCNTGCGCNG ANCCNCAGCG NCCNGTNATN
Conservation
crp_native AGGCTGCATC CCGTGACGGC CGACGACGCG CCGAAGCTGC CGGCGACCCG CTGCTCGACC 120
crp_altered CGCCTCCACC CGGTCACCGC GGATGATGCC CCCAAGCTTC CCGCCACGCG GTGCAGCACG 120
Consensus NGNCTNCANC CNGTNACNGC NGANGANGCN CCNAAGCTNC CNGCNACNCG NTGCNNNACN
Conservation
crp_native TGCGCGATAC GCAACGTGTG CATGCCGGCC GGCCTCACGC CGATCGAATT CGCGCGGCTC 180
crp_altered TGCGCAATCA GGAATGTCTG CATGCCCGCG GGGCTGACCC CCATTGAGTT CGCCCGCCTG 180
Consensus TGCGCNATNN GNAANGTNTG CATGCCNGCN GGNCTNACNC CNATNGANTT CGCNCGNCTN
Conservation
crp_native GACGCGATGG TCTGCACGAC GCGCCACGTC AAGCGCGGCG ACGCGCTGTT CCGCACGCAC 240
crp_altered GATGCCATGG TGTGCACCAC CCGGCACGTG AAGCGAGGGG ATGCCCTCTT CCGGACCCAC 240
Consensus GANGCNATGG TNTGCACNAC NCGNCACGTN AAGCGNGGNG ANGCNCTNTT CCGNACNCAC
Conservation
crp_native GACACGTTCC AGAGCATCTA TGCGGTGCGC ACCGGCTCGT TCAAGACGGT CGTCATGCAC 300
crp_altered GATACCTTCC AGTCGATTTA CGCCGTCCGG ACGGGGAGCT TCAAGACCGT GGTGATGCAC 300
Consensus GANACNTTCC AGNNNATNTA NGCNGTNCGN ACNGGNNNNT TCAAGACNGT NGTNATGCAC
Conservation
crp_native CGCGACGGCC GCGAGCAGGT AACGGGCTTC CAGATCGTCG GCGAGACGCT CGGGCTGGAC 360
crp_altered CGGGATGGGC GGGAACAGGT GACCGGGTTC CAGATTGTGG GGGAAACCCT GGGCCTGGAT 360
Consensus CGNGANGGNC GNGANCAGGT NACNGGNTTC CAGATNGTNG GNGANACNCT NGGNCTGGAN
Conservation
crp_native GGCGTGTGCG GCGGCCGCCA CAACAGCGAT GCGATTGCGC TCGAGGACAG CACCGTGTGC 420
crp_altered GGGGTCTGCG GGGGGCGTCA CAATTCTGAC GCCATCGCGC TGGAAGATTC GACGGTTTGC 420
Consensus GGNGTNTGCG GNGGNCGNCA CAANNNNGAN GCNATNGCNC TNGANGANNN NACNGTNTGC
Conservation
crp_native ATCATCCCGT TCGCGCAGCT CGAGGCCGTG TGCCGCGAGG TGAAGCCGAT GCAGCACCAC 480
crp_altered ATTATTCCCT TCGCCCAGCT GGAAGCAGTT TGCCGGGAAG TCAAGCCCAT GCAGCATCAT 480
Consensus ATNATNCCNT TCGCNCAGCT NGANGCNGTN TGCCGNGANG TNAAGCCNAT GCAGCANCAN
Conservation
crp_native GTGCATCAGT TGATGAGCGG CGAGATCGTT CGCGAATCGA GCCTGATGAT GCTGCTCGGC 540
crp_altered GTCCACCAGC TCATGTCCGG GGAAATTGTG CGGGAGAGCT CGCTCATGAT GCTCCTGGGG 540
Consensus GTNCANCAGN TNATGNNCGG NGANATNGTN CGNGANNNNN NNCTNATGAT GCTNCTNGGN
Conservation
crp_native ACGATGACGG CCGAGCAGCG CGTTGCCGCA TTCCTGCTCA ATATTTCCGA GCGTTTCCAG 600
crp_altered ACCATGACCG CGGAACAGCG GGTGGCGGCG TTCCTCTTGA ACATCAGCGA ACGCTTCCAG 600
Consensus ACNATGACNG CNGANCAGCG NGTNGCNGCN TTCCTNNTNA ANATNNNCGA NCGNTTCCAG
Conservation
crp_native AAGCGCGGCT ATTCGGCATC GGAATTCAAC CTGCGAATGA CGCGCGAGGA AATCGGCTGC 660
crp_altered AAGCGGGGGT ACAGCGCGAG CGAGTTCAAT CTCCGCATGA CCCGGGAAGA GATTGGGTGC 660
Consensus AAGCGNGGNT ANNNNGCNNN NGANTTCAAN CTNCGNATGA CNCGNGANGA NATNGGNTGC
Conservation
crp_native TACCTCGGGA TGAAGCTCGA GACGGTCAGC CGGATGCTGT CGAAGTTCCA GCGCGACCGG 720
crp_altered TATCTGGGCA TGAAGCTGGA AACCGTGTCG CGCATGCTCT CGAAGTTCCA GCGGGATCGC 720
Consensus TANCTNGGNA TGAAGCTNGA NACNGTNNNN CGNATGCTNT CGAAGTTCCA GCGNGANCGN
Conservation
crp_native TTGATCGCGC CGCGCGGCAA GCAGATCCGC ATCACCGATC CGCAGGGGCT GGCGCGCGTG 780
crp_altered CTCATTGCAC CCCGGGGGAA GCAGATTCGG ATTACGGACC CCCAGGGCTT GGCCCGGGTA 780
Consensus NTNATNGCNC CNCGNGGNAA GCAGATNCGN ATNACNGANC CNCAGGGNNT NGCNCGNGTN
Conservation
crp_native TGA 783
crp_altered TGA 783
Consensus TGA
Conservation
c
ComE_native_AA MLKKLLATAA LCAAAAHGWA AVDVNAANDD ALRGIRGIGP AKAKAIVDER SAHGPFKDAA 60
ComE_altered_AA MLKKLLATAA LCAAAAHGWA AVDVNAANDD ALRGIRGIGP AKAKAIVDER SAHGPFKDAA 60
Consensus MLKKLLATAA LCAAAAHGWA AVDVNAANDD ALRGIRGIGP AKAKAIVDER SAHGPFKDAA
Conservation
ComE_native_AA DLARRVKGMG GKTVERLQAE GLAIGAARAP AAAGAPQKAA AAGKK 105
ComE_altered_AA DLARRVKGMG GKTVERLQAE GLAIGAARAP AAAGAPQKAA AAGKK 105
Consensus DLARRVKGMG GKTVERLQAE GLAIGAARAP AAAGAPQKAA AAGKK
Conservation
d
Crp_native_AA MLTASVNEAE PCAEPQRPVI RLHPVTADDA PKLPATRCST CAIRNVCMPA GLTPIEFARL 60
Crp_altered_AA MLTASVNEAE PCAEPQRPVI RLHPVTADDA PKLPATRCST CAIRNVCMPA GLTPIEFARL 60
Consensus MLTASVNEAE PCAEPQRPVI RLHPVTADDA PKLPATRCST CAIRNVCMPA GLTPIEFARL
Conservation
Crp_native_AA DAMVCTTRHV KRGDALFRTH DTFQSIYAVR TGSFKTVVMH RDGREQVTGF QIVGETLGLD 120
Crp_altered_AA DAMVCTTRHV KRGDALFRTH DTFQSIYAVR TGSFKTVVMH RDGREQVTGF QIVGETLGLD 120
Consensus DAMVCTTRHV KRGDALFRTH DTFQSIYAVR TGSFKTVVMH RDGREQVTGF QIVGETLGLD
Conservation
Crp_native_AA GVCGGRHNSD AIALEDSTVC IIPFAQLEAV CREVKPMQHH VHQLMSGEIV RESSLMMLLG 180
Crp_altered_AA GVCGGRHNSD AIALEDSTVC IIPFAQLEAV CREVKPMQHH VHQLMSGEIV RESSLMMLLG 180
Consensus GVCGGRHNSD AIALEDSTVC IIPFAQLEAV CREVKPMQHH VHQLMSGEIV RESSLMMLLG
Conservation
Crp_native_AA TMTAEQRVAA FLLNISERFQ KRGYSASEFN LRMTREEIGC YLGMKLETVS RMLSKFQRDR 240
Crp_altered_AA TMTAEQRVAA FLLNISERFQ KRGYSASEFN LRMTREEIGC YLGMKLETVS RMLSKFQRDR 240
Consensus TMTAEQRVAA FLLNISERFQ KRGYSASEFN LRMTREEIGC YLGMKLETVS RMLSKFQRDR
Conservation
Crp_native_AA LIAPRGKQIR ITDPQGLARV 260
Crp_altered_AA LIAPRGKQIR ITDPQGLARV 260
Consensus LIAPRGKQIR ITDPQGLARV
Conservation
